# Endothelial-specific ablation of Serum Response Factor causes hemorrhaging, yolk sac vascular failure, and embryonic lethality

**DOI:** 10.1186/1471-213X-8-65

**Published:** 2008-06-20

**Authors:** Mary L Holtz, Ravi P Misra

**Affiliations:** 1 Department of Biochemistry, Medical College of Wisconsin, Milwaukee, Wisconsin, USA

## Abstract

**Background:**

Serum response factor (SRF), a member of the MADS box family of nuclear transcription factors, plays an important role in cardiovascular development and function. Numerous studies demonstrate a central role for SRF in regulating smooth and cardiac muscle cell gene expression. Consistent with this, loss of SRF function blocks differentiation of coronary vascular smooth muscle cells from proepicardial precursors, indicating SRF is necessary for coronary vasculogenesis. The role of SRF in endothelial cell contribution during early vascular development, however, has not been addressed. To investigate this, we generated transgenic mice lacking expression of SRF in endothelial cells. Mice expressing Cre recombinase (*Tie2Cre*^+^) under *Tie2 *promoter control were bred to mice homozygous for *Srf *alleles containing loxP recombination sites within the *Srf *gene (*Srf*^*f*/*f*^). Tie2 is a tyrosine kinase receptor expressed predominantly on endothelial cells that mediates signalling during different stages of blood vessel remodelling. Resulting embryos were harvested at specific ages for observation of physical condition and analysis of genotype.

**Results:**

*Tie2Cre*^+/-^*Srf*^*f*/*f *^embryos appeared to develop normally compared to wild-type littermates until embryonic day 10.5 (E10.5). Beginning at E11.5, *Tie2Cre*^+/-^*Srf*^*f*/*f *^embryos exhibited cerebrovascular hemorrhaging and severely disrupted vascular networks within the yolk sac. Hemorrhaging in mutant embryos became more generalized with age, and by E14.5, most *Tie2Cre*^+/-^*Srf*^*f*/*f *^embryos observed were nonviable and grossly necrotic. Hearts of mutant embryos were smaller relative to overall body weight compared to wild-type littermates. Immunohistochemical analysis revealed the presence of vascular endothelial cells; however, vessels failed to undergo appropriate remodelling. Initial analysis by electron microscopy suggested a lack of appropriate cell-cell contacts between endothelial cells. Consistent with this, disrupted E-cadherin staining patterns were observed in mutant embryos.

**Conclusion:**

These results provide the first *in vivo *evidence in support of a role for SRF in endothelial cell function and strongly suggest SRF is required for appropriate vascular remodelling.

## Background

Serum response factor (SRF) is a nuclear transcription factor of the MADS (MCM1, Agamous, Deficiens, SRF) box family. SRF interacts as a dimer with DNA at the serum response element (SRE), a 10 base pair AT-rich sequence [CC(AT)_6_GG] also known as the CArG box (for review see [1,2]). The SRE sequence is present in a wide variety of genes, including those encoding for immediate early proteins (e.g. Fos, Jun, HSP70), neuronal nuclear receptors (e.g. Nurr1, Nur77) and numerous contractile and cytoskeletal proteins (eg. actins, myosins). Expression of SRF is essential in early development as SRF-null embryos die during gastrulation [3]. SRF is required for development of mesoderm [3]. The expression pattern of genes under SRF control is regulated in a combinatorial fashion by SRF's ability to interact with a variety of accessory factors such as Elk-1 and SAP-1 to regulate expression of genes involved in cell growth and proliferation, and myocardin and related family factors to control myogenic gene expression (for review see [4]).

SRF plays a critical role in myogenesis. Early studies established the expression of SRF in cells of the myogenic lineage [5-7], and SRF has been shown to be required for appropriate skeletal, cardiac and smooth muscle cell growth and differentiation [8,9]. SRF gene knockout studies from our laboratory indicate that disruption of SRF in cardiomyocytes leads to severe defects in the contractile apparatus, including Z-disc and stress fiber formation, as well as mislocalization and/or attenuation of sarcomeric protein expression [10]. Consistent with these observations, *in vivo *cardiovascular-specific knockout of SRF results in embryonic death via cardiovascular failure due to perturbations in normal muscle cytoarchitecture and contractile assembly [11-13]. Numerous studies suggest that disordered myogenic gene expression is due to defective SRF-mediated regulation of the smooth muscle cell-specific regulatory factor myocardin [14-16].

SRF is a key regulator in development of the coronary vasculature. Coronary vasculature is derived from a transient embryonic structure termed the proepicardium (PE) [17-19]. The PE is characterized as a small, grape cluster-like aggregate of cells developing at embryonic day 9 (E9) from an extension of the septum transversum, lying beneath the developing heart tube [20]. Cells of the PE undergo an epithelial-to-mesenchymal transition (EMT) and migrate over the surface of the heart. Some cells form the primitive epicardium, and others appear to continue to infiltrate into the subepicardial space. Once dispersed into the heart tissue, these subepicardial mesenchymal cells (SEMC) differentiate into the vascular smooth muscle cells (VSMC) and vascular endothelial cells (VEC) that comprise the coronary vasculature [18]. Previous work in our laboratory has demonstrated expression of SRF within cells of the PE and newly formed coronary vessels [21]. Landerholm and colleagues [22] have shown that inhibitory SRF constructs block differentiation of avian PE to VSMC, indicating a central role for SRF in differentiation of PE-derived cells to VSMC.

A role for SRF in differentiation and function of VSMC is well established, however, little is known about the role of SRF in VEC [5,23]. Using primary HUVEC cultures, Chai and colleagues [24] have shown that vascular endothelial growth factor (VEGF) signalling pathways require SRF. Endothelial cells in which SRF has been knocked down fail to respond to intercellular VEGF signalling and show abolished VEGF-induced *in vitro *angiogenesis, impaired endothelial cell migration and proliferation, and inhibited VEGF-induced actin polymerization and immediate early gene expression.

These studies indicate that SRF likely plays an important role in endothelial cell function. The *in vivo *role of endothelial SRF, however, has not been addressed. Therefore, in the current study we investigated the role of SRF in VEC function by carrying out endothelial cell-specific ablation of the SRF gene. Mice homozygous for *Srf*-Lox P (*Srf*^*f*/*f*^) alleles were bred to mice expressing the Cre recombinase protein under *Tie2 *promoter control (*Tie2Cre*^+/-^). Tie2 is a vascular endothelial-specific tyrosine kinase receptor for the angiopoietin family of vascular remodelling factors [25,26]. The onset of *Tie2Cre *expression is concurrent with the appearance of endothelium in E7.5 embryos and continues in adult tissues [27]. In embryos expressing the mutant genotype *Tie2Cre*^+/-^*Srf*^*f*/*f*^, we observed hemorrhaging by E11.5 and near complete lethality by E14.5. Examination of cardiac morphology revealed smaller hearts as well as mild ventricular-septal defects in mutant embryos as compared to wild-type littermates. Immunohistochemical analysis suggests VEC properly differentiated and assembled blood vessels. However, the appearance of blood pools and segmented blood vessels observed in embryos and yolk sacs of E11.5–E13.5 embryos indicated disrupted vascular networks consistent with a defect in vascular remodelling. Further analysis of yolk sac tissues by electron microscopy demonstrated a lack of desmosomal-type junction complexes as well as a paucity of collagen matrix between the endodermal and mesodermal cell layers. E-cadherin protein detected by immunofluorescence showed a dramatic decrease in signal along the apical brush-border of endothelial cells in the extraembryonic endodermal layer in yolk sac. These results provide the first *in vivo *evidence in support of a role for SRF in endothelial cell function and strongly suggest SRF is required for appropriate vascular remodelling.

## Results

### Conditional ablation of SRF protein in endothelium causes hemorrhaging in developing embryos

**Figure 1:**
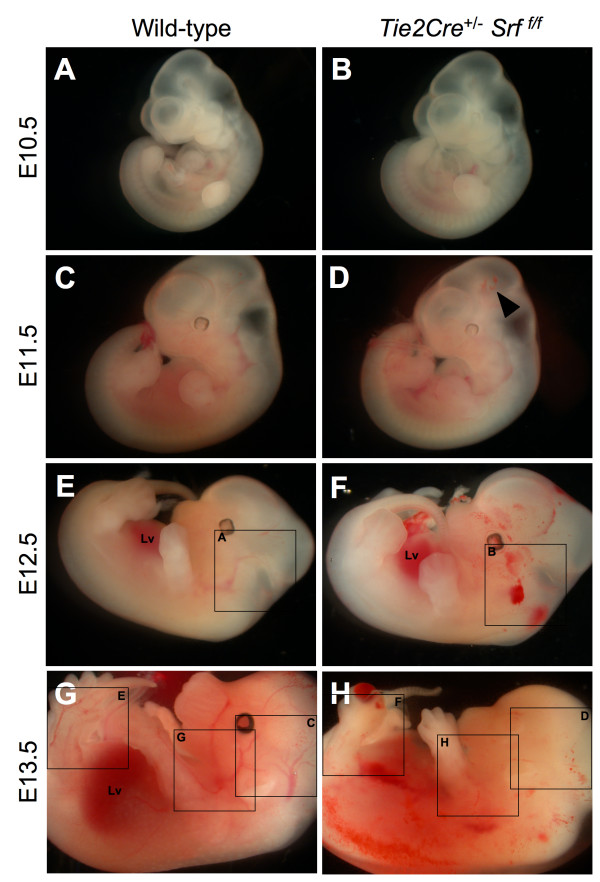
***Tie2Cre*^+/-^*Srf*^*f*/*f *^embryos exhibit internal hemorrhaging**. Photographs of whole unfixed, unstained mouse embryos at E10.5 (A, B), E11.5 (C, D), E12.5 (E, F) and E13.5 (G, H). *Tie2Cre*^+/-^*Srf*^*f*/*f *^mutant embryos (B, D, F, H) exhibited internal hemorrhaging compared to wild-type littermates (A, C, E, G). This effect progressed with increasing gestational age. E14.5 mutant embryos were grossly necrotic and unsuitable for analysis. Labelled boxes in Fig. 1 correspond to images in Fig. 2.; Lv = liver. Arrowhead marks minimal hemorrhaging observed in E11.5 embryos.

**Figure 2:**
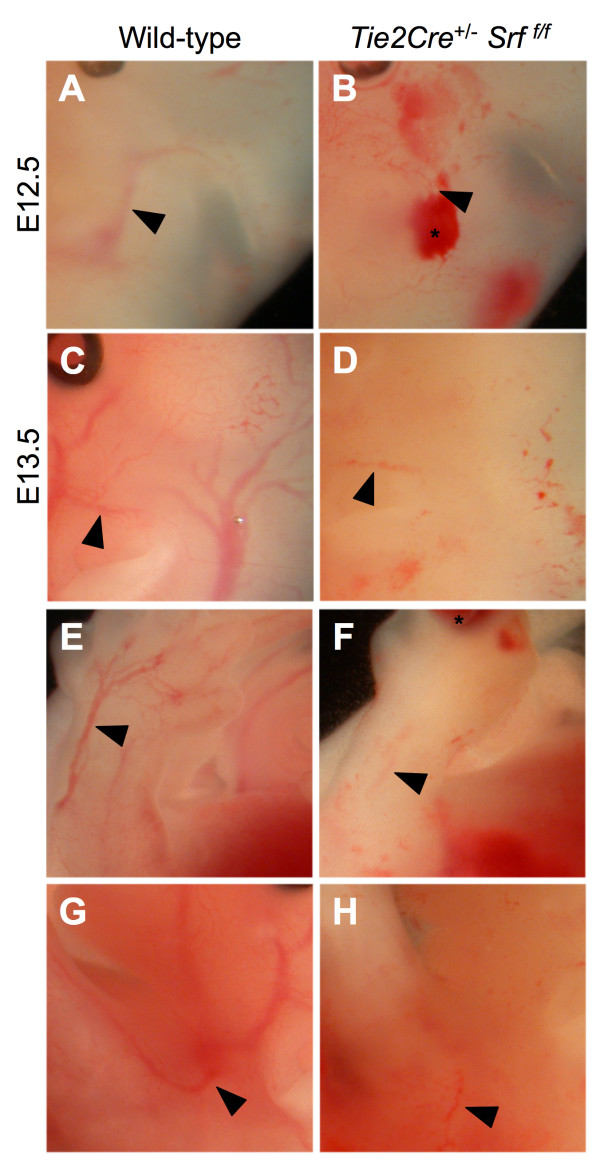
**Vascular fragmentation and internal hemorrhaging in *Tie2Cre*^+/-^*Srf*^*f*/*f *^embryos**. Higher magnification photographs of whole unfixed, unstained mouse embryos as in Figure 1; embryos were examined at E12.5 (A, B) and E13.5 (C-H). *Tie2Cre*^+/-^*Srf*^*f*/*f *^mutant embryos (B, D, F, H) exhibited blood pooling (asterisks) and loss of visible vascular structures (arrowheads mark similar vessels; compare A & B, C & D, E & F, and G & H).

Inspection of embryos harvested from *Tie2Cre*^+/-^*Srf*^*f*/+ ^× *Srf*^*f*/*f *^matings revealed internal hemorrhaging and blood pooling consistent with loss of vascular organization (see Figures 1 and 2). Mutant embryos appeared to develop normally until E10.5 (compare Fig. 1A and 1B). However, by E11.5 embryos expressing the mutant genotype *Tie2Cre*^+/-^*Srf*^*f*/*f *^began to exhibit internal hemorrhaging (Fig. 1D, arrowhead). The extent of bleeding continued to progress with embryonic age (Fig. 1D, F, &1H). Wild-type littermates showed intact vascular formation in contrast to mutant embryos (compare Fig. 1G and 1H). Upon closer examination, loss of vascular formation in the mutant embryos becomes more obvious (see Figure 2). A striking difference in the lack of apparent intact vasculature was observed in *Tie2Cre*^+/-^*Srf*^*f*/*f *^embryos compared to wild-type littermates (Fig. 2, compare arrows in A and B, C and D, E and F, and G and H). Blood pooling noted in mutant embryos (Fig. 2B and 2F, asterisks) supports a loss of vascular integrity.

### Endothelial-specific disruption of the *Srf *gene during development causes embryonic lethality

The effect of endothelial-specific conditional ablation of SRF protein expression during embryonic development was evaluated. Loss of SRF expression in endothelial cells was achieved by Tie2 driven-Cre recombinase-mediated excision of the promoter region and first exon of *Srf *[11]. The conditional loss of *Srf *gene function in endothelial cells resulted in embryonic death by E14.5 (see Table 1). The majority of animals expressing the mutant genotype *Tie2Cre*^+/-^*Srf*^*f*/*f *^displayed an abnormal phenotype, and all necrotic embryos collected expressed the mutant genotype. No animals with a mutant genotype were detected in late gestation or at birth.

**Table 1:** Embryos with mutant genotype *Tie2·Cre*^+/-^*Srf*^*f*/*f *^die by E14.5

**Age**	***Cre*^+^/*Srf***^*f*/*f*^	***Cre*^+^/*Srf***^*f*/+^	***Cre*^-^/*Srf***^*f*/*f*^	***Cre*^-^/*Srf***^*f*/+^
E9.5	7 (0)	5	10	16
E10.5	12 (0)	14 (1)	15	26
E11.5	19 (1)	29 (2)	7	17
E12.5	24 (11)	21	17	7
E13.5	21 (19)	15	14 (2)	16
E14.5	10 (8)	3	3	4
E18.5	2 (2)	4	2	5
Neonate	0	18	20	13

Genotypic analysis of mouse embryos generated by crossing *Tie2Cre*^+/-^*Srf*^*f*/+ ^male mice with *Srf*^*f*/*f *^female mice. Numbers represent total embryos of each genotype; numbers in parentheses indicate mutant phenotype and grossly necrotic embryos. The conditional loss of *Srf *gene function in endothelial cells resulted in embryonic death by E14.5; no animals with mutant genotype were detected during late gestational age or at birth.

### Hearts of *Tie2Cre*^+/-^*Srf*^*f/f *^mutant embryos are smaller

**Figure 3:**
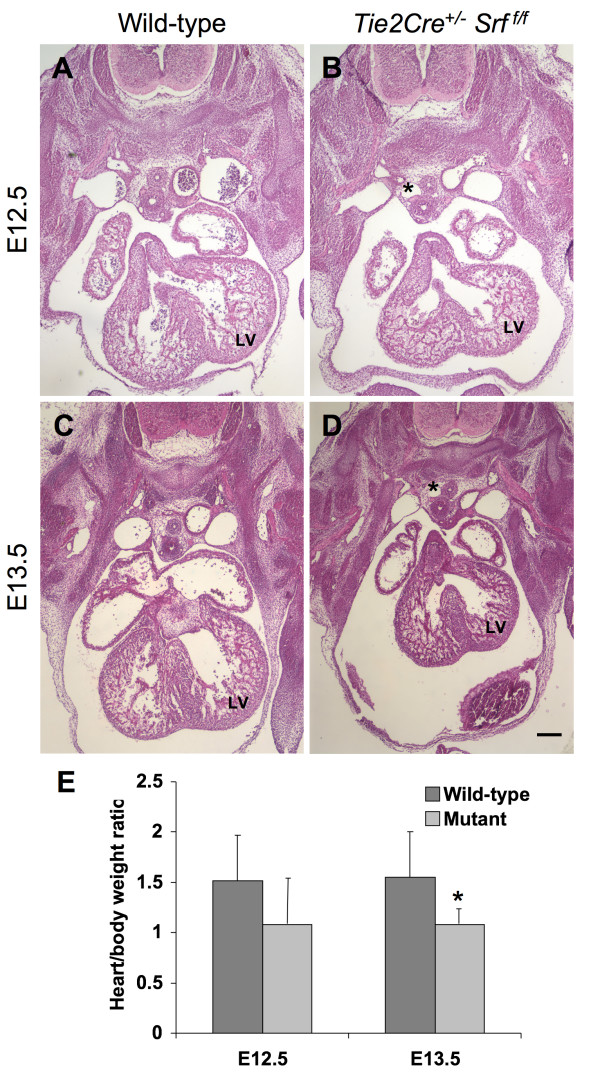
**Hearts in mutant embryos are smaller and dysmorphic**. Photomicrographs (40×) of transverse sections of mouse embryos at E12.5 (A, B) and E13.5 (C, D) stained with hematoxylin and eosin. *Tie2Cre*^+/-^*Srf*^*f*/*f *^embryos exhibited reduced heart size by E12.5 (B, compare to A), which became more pronounced and consistent at E13.5 (D, compare to C). (E) Quantitation of heart size normalized to overall body weight revealed smaller hearts in both E12.5 (1.5 ± 0.4 vs. 1.08 ± 0.5, wild-type vs. mutant respectively; p = 0.054) and E13.5 embryos (1.4 ± 0.39% vs. 1.04 ± 0.17%, wild-type vs. mutant respectively; p = 0.021). Enlarged vessels apparently caused by edema due to vascular insufficiency were also noted in mutant embryos (asterisks). LV = left ventricle. Scale bar: 200 μm.

Since SRF plays a central role in cardiac development, we examined cardiac morphology of mutant embryos and their wild-type littermates to ascertain whether observed embryonic lethality was due to abnormal cardiac morphogenesis. Embryos harvested from timed-pregnant female mice were subjected to fixation for analysis of morphology or for quantification of whole body and isolated heart weights. Hearts of wild-type embryos correctly formed atrial and ventricular chambers, and major vessels of the atrial tree were present (see Figure 3). In contrast, the muscular portion of the interventricular septum in hearts of *Tie2Cre*^+/-^*Srf*^*f*/*f *^mutant genotype embryos appeared reduced (Fig. 3B and 3D), potentially resulting in ventricular-septal defects. Furthermore, enlarged vessels were observed in mutant embryos, presumably due to edema caused by vascular insufficiency (Fig. 3B and 3D, asterisks). Analysis of endocardial cushion extracellular matrix deposition revealed no difference between wild-type and mutant littermates (alcian blue staining; data not shown).

Hearts of mutant embryos appeared smaller than those of wild-type littermates. To assess this observation, whole body and isolated heart weights from E12.5 and E13.5 embryos were used to calculate heart weights as a percentage of overall body weight (Fig. 3E). The decrease in apparent size of mutant hearts appeared as early as E12.5 (compare Fig. 3A with 3B); however, differences in whole body and heart weights at this age did not reach statistical significance (1.5 ± 0.4 vs. 1.08 ± 0.5, wild-type vs. mutant respectively; p = 0.054). Normalized heart weights from E13.5 mutant embryos were reduced (1.4 ± 0.39% vs. 1.04 ± 0.17%, wild-type vs. mutant respectively; p = 0.021). Furthermore, immunofluorescent analysis of phosphorylated histone H3 in E11.5 embryos revealed an apparent reduction in the number of endocardial endothelial cells undergoing proliferation (see Additional file 1); no difference in apoptotic activity was detected (cleaved caspase 3 immunofluorescent analysis; data not shown). In contrast, the ratio of cardiomyocytes to total cells was not different between wild-type and mutant heart tissues (MF-20-immunolabelled cells as a percentage of total DAPI-positive nuclei; data not shown), suggesting that the heart size differences noted are not due to a selective loss of cardiomyocytes. E14.5 embryos with mutant genotype were grossly necrotic and unsuitable for analysis.

### Yolk sac vasculature fails in *Tie2Cre*^+/-^*Srf*^*f/f *^mutant embryos

**Figure 4:**
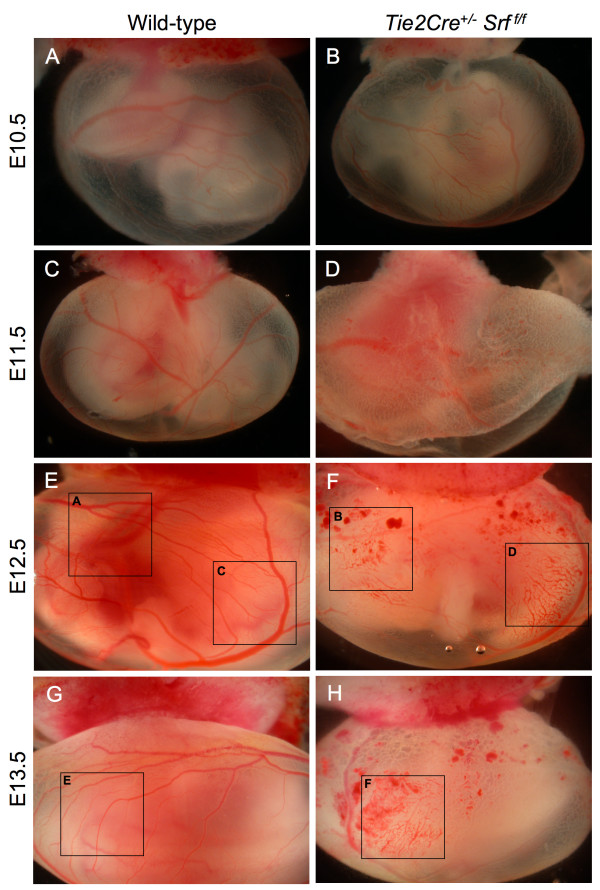
***Tie2Cre*^+/-^*Srf*^*f*/*f *^embryos exhibit yolk sac vascular failure**. Photographs of whole unfixed, unstained intact yolk sacs containing mouse embryos at E10.5 (A, B), E11.5 (C, D), E12.5 (E, F) and E13.5 (G, H). Embryos with *Tie2Cre*^+/-^*Srf*^*f*/*f *^genotype exhibited severely disrupted vascular networks at E12.5 (F) and E13.5 (H) as compared to wild-type littermates (E and G respectively). Labelled boxes in Fig. 4 correspond to images in Fig. 5.

**Figure 5:**
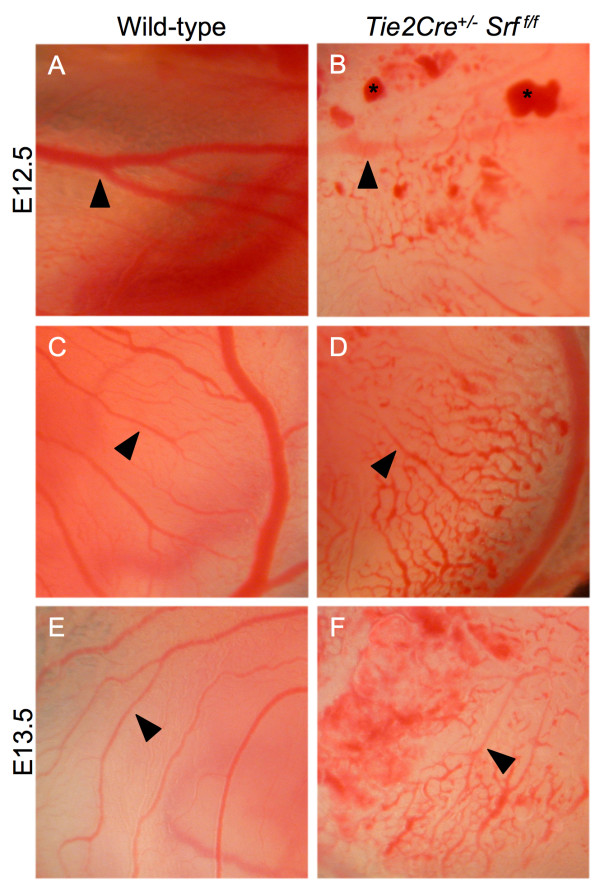
**Yolk sac tissues of *Tie2Cre*^+/-^*Srf*^*f*/*f *^embryos display vascular disintegration and blood pooling**. Photographs of whole unfixed, unstained intact yolk sacs containing mouse embryos at E12.5 (A, B, C, D) and E13.5 (E, F). Yolk sac tissues from mutant embryos showed disorganized vascular networks at E12.5 (B) that become more pronounced at E13.5 (D, F arrowheads). Blood pools (B, asterisks) were also observed in mutant yolk sac tissues.

The *Tie2Cre *construct is expressed in blood islands and hemangioblasts within the extraembryonic yolk sac as early as E7.5 [27]. Ablation of SRF protein expression in yolk sac endothelial tissues caused blood vessel disorganization and subsequent vascular failure (see Figures 4 and 5). Yolk sac blood vessels appeared normal in both wild-type and mutant animals until E11.5 (compare Fig. 4A and 4B, C and 4D). In contrast, the yolk sacs of E12.5 and E13.5 mutant embryos contained blood pools and fragmented blood vessels (Fig. 4F and 4H), consistent with impaired vascular remodelling. Closer examination revealed a web of poorly organized vessels (Fig. 5, compare arrowheads in 5A, C, and 5E with 5B, D, and 5F).

### SRF protein is localized within endothelial cells

**Figure 6:**
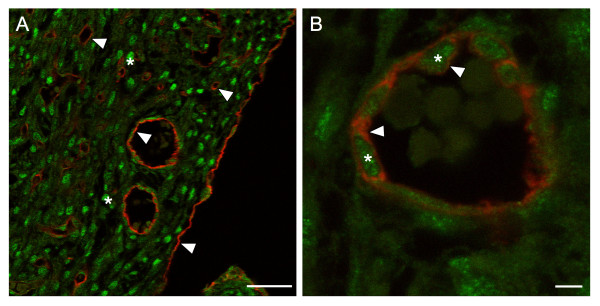
**Detection of SRF protein within vascular endothelial cells**. Single-plane confocal laser photomicrographs (A-630×, B-1,000×) of ventricular tissue from wild-type E16.5 mouse heart immunolabelled with the VEC marker PECAM-1 (red) and SRF (green). (A) PECAM-1-stained cells surround vessels as well as form the epicardium (arrowheads). Cardiomyocytes within ventricular tissue show strong SRF staining (asterisks). (B) Endothelial cell-specific PECAM-1 (red; arrowheads) encircles the nuclear-localized SRF protein (green; asterisks). Scale bars: A = 40 μm; B = 4 μm.

To establish the expression of SRF within VEC of the coronary vasculature, we performed double-label immunofluorescent analysis of embryonic mouse heart. Ventricular tissue was isolated from wild-type E16.5 mouse embryos, after coronary vessels have formed; corresponding *Tie2Cre*^+/-^*Srf*^*f*/*f *^samples were not obtainable as affected embryos die by E14.5. Sections of paraffin-embedded ventricular tissue were immunolabelled for both PECAM-1 and SRF proteins and examined by confocal laser microscopy (see Figure 6). PECAM-1-stained endothelial cells were clearly visible surrounding vessels in ventricular myocardium as well as surrounding the myocardial wall (Fig. 6A, arrowheads). Cardiomyocytes showed robust SRF staining (Fig. 6A, asterisks). Higher magnification imaging of a single focal plane showed nuclear-localized SRF immunostaining (Fig. 6B, asterisks) surrounded by PECAM-1 labelled endothelial cell membranes (Fig. 6B, arrowheads).

### SRF protein is decreased in VEC from *Tie2Cre*^+/-^*Srf*^*f/f *^embryos

**Figure 7:**
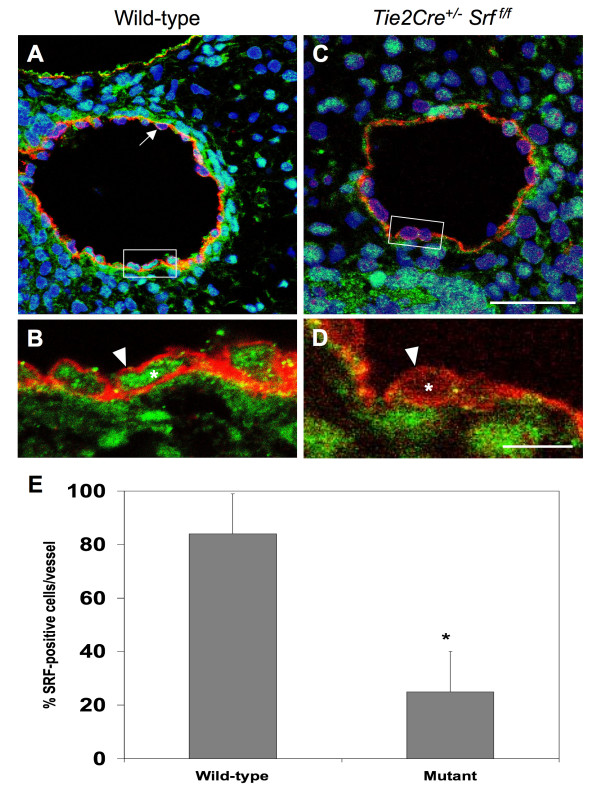
**SRF protein is decreased in endothelial tissues of *Tie2Cre*^+/-^*Srf*^*f*/*f *^embryos**. Photomicrographs (A, C:1,000×; B, D:4,000×) of cross-sections of blood vessels from E10.5 wild-type (A, B) and mutant (C, D) embryos were used to count the total number of VEC and the number of VEC exhibiting SRF (boxes in A, C correspond to images in C, D respectively). Tissues were labelled with antibodies against SRF (green) and the VEC-marker PECAM-1 (red) and analyzed using single-plane confocal laser microscopy (blue = Topro3 nuclear staining). Only those cells showing SRF signal completely encircled by PECAM-1 staining were scored as SRF-positive VEC (B, D arrowheads). Not all VEC displayed detectable SRF protein (A, arrow). Final numbers were expressed as percent SRF-positive VEC of the total counted for that vessel (E): 84 ± 14.3 vs. 25 ± 15.7, wild-type vs. mutant respectively; *p = 0.0000004, student's *t *test. Scale bars: A, C = 30 μm; B, D = 10 μm.

We analyzed embryonic vascular tissues using double-label immunofluorescence microscopy to verify the loss of SRF protein in affected cells (see Figure 7). Tissues from E10.5 wild-type (Fig. 7A and 7B) and mutant (Fig. 7C and 7D) embryos were labelled with antibodies against SRF and the VEC-marker PECAM-1 and analyzed by confocal laser microscopy. Single-plane confocal images of blood vessels were used to count the total number of VEC and the number of VEC exhibiting SRF. Only those cells showing SRF signal completely encircled by PECAM-1 staining were scored as SRF-positive VEC (Fig. 7B, arrowhead). Final numbers were expressed as percent SRF-positive VEC of the total counted for that vessel. A high proportion of VEC in wild-type vessels were positive for SRF (84% ± 14.3) compared to mutant vessels (25% ± 15.7; p < 0.0000004; Fig. 7E).

Since the *Tie2Cre *construct has been shown to be expressed in endocardial endothelial cells, we analyzed embryonic ventricular tissue for the presence or absence of SRF protein using single-plane confocal images as described above (see Additional file 2). We found a higher number of endocardial endothelial cells exhibiting SRF-positive immunofluorescence in wild-type as compared to mutant tissues. No significant change in expression of SRF protein was observed between wild-type or mutant embryos in the epicardium (Additional file 2). Furthermore, to verify that SRF protein expression in cardiomyocytes was not grossly affected by loss of SRF in endothelial cells, we analyzed SRF protein expression in embryonic heart tissues by immunodetection; however, no differences were noted (Additional file 2).

### Early coronary vessel formation appears normal in mutant embryos

**Figure 8:**
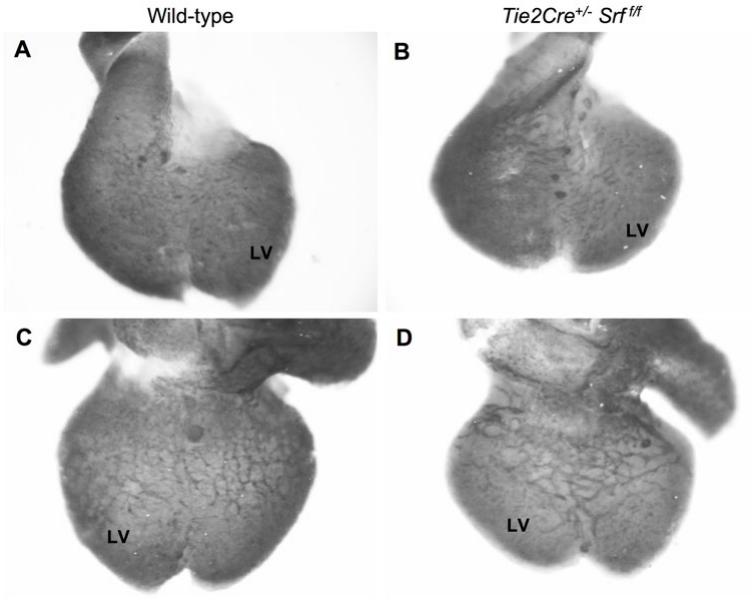
**Early coronary vessel development in *Tie2Cre*^+/-^*Srf*^*f*/*f *^embryos appears normal compared to wild-type littermates**. Photographs of whole-mount immunostained wild-type (A, C) and *Tie2Cre*^+/-^*Srf*^*f*/*f *^(B, D) hearts. Samples were stained with the endothelial cell marker PECAM-1 to detect vascular structures. Vessels on ventral (A, B) and dorsal (C, D) heart surfaces were clearly visible, indicating apparently normal assembly of initial coronary vasculature. LV = left ventricle.

Coronary vessels begin to form at approximately E11.5, with the coronary vascular network complete by E16.5 [28]. We examined early coronary vessel formation by whole-mount immunohistochemical analysis of hearts from E12.5 embryos (see Figure 8). We used the VEC-specific cell-surface marker PECAM-1 to label vascular structures in hearts from both wild-type and *Tie2Cre*^+/-^*Srf*^*f*/*f *^embryos. Vessels on the external ventral (Fig. 8A and 8B) and dorsal (Fig. 8C and 8D) surfaces are visible and seem intact in both wild-type and *Tie2Cre*^+/-^*Srf*^*f*/*f *^embryos. This observation indicates that initial formation of the coronary vasculature is not affected by loss of SRF in endothelial cells. However, we were unable to complete assessment of the angiogenic remodelling responsible for maturation of the coronary vascular network in mutant embryos as they die and become grossly necrotic by E14.5.

### Yolk sac tissues from *Tie2Cre*^+/-^*Srf*^*f/f *^embryos retain VEC despite fragmentation of blood vessels

**Figure 9:**
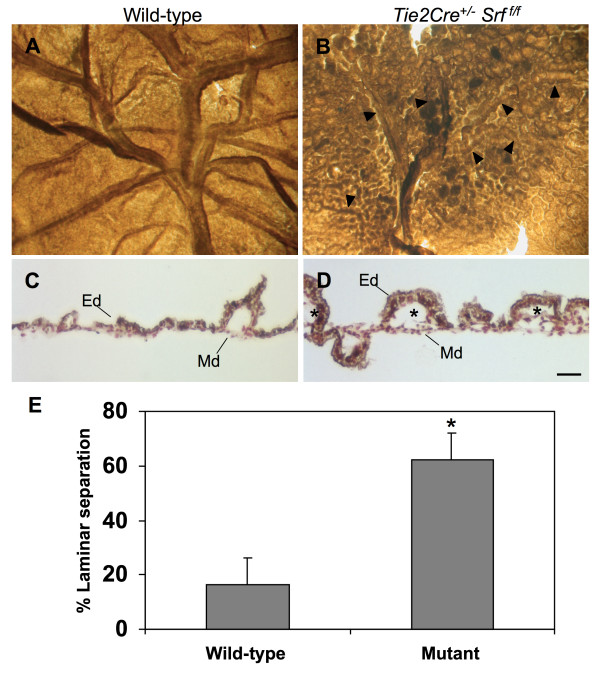
***Tie2Cre*^+/-^*Srf*^*f*/*f *^yolk sac tissues show loss of blood vessel integrity and separation of dermal layers**. Yolk sac tissues immunostained with VEC-specific marker PECAM-1 to label endothelial cells and vascular structures. Photographs of wild-type (A) and *Tie2Cre*^+/-^*Srf*^*f*/*f *^(B) whole yolk sac at E13.5; arrowheads (B) mark apparent blood vessel remnants in mutant yolk sac tissue. Photomicrographs (200×) of cross-sections of wild-type (C) and *Tie2Cre*^+/-^*Srf*^*f*/*f *^(D and E) yolk sac at E13.5 immunolabelled for PECAM-1 (brown-gray = PECAM-1, red = neutral red counterstain). Normal cellular layers of endoderm (Ed) and extraembryonic mesoderm (Md) are tightly associated in wild-type as compared to mutant yolk sac tissue. Asterisks mark areas of inappropriate tissue layer separation in *Tie2Cre*^+/-^*Srf*^*f*/*f *^yolk sac (D). (E) Quantitation of laminar separation in cross-sections of yolk sac tissues as in C and D revealed increased delamination in mutant animals (16.3 ± 10.8% vs. 62.0 ± 10.4%, wild-type vs. mutant respectively; p = 0.0031). Scale bar: C, D = 50 μm.

The *Tie2Cre *construct is expressed in endodermal tissue layers of the yolk sac as well as in VEC. Yolk sac vascular networks undergo extensive remodelling during the course of embryogenesis. Based on our observation of fragmented yolk sac vessels, we sought to determine the status of VEC within yolk sac tissues. We looked for the presence of VEC within yolk sac tissues by immunolabelling whole mount samples of E13.5 wild-type and *Tie2Cre*^+/-^*Srf*^*f*/*f *^yolk sac tissues with the VEC marker PECAM-1 (see Figure 9). Yolk sac tissues from embryos lacking SRF in endothelium demonstrated the presence of appropriately differentiated VEC; however, blood vessels in SRF-null yolk sac tissues were almost completely disintegrated by this age. Remnants of presumed blood vessels could still be seen (Fig. 9B, arrowheads), suggesting incomplete vessel remodelling or inappropriate maintenance of existing vessels.

Furthermore, cross-sectional examination of *Tie2Cre*^+/-^*Srf*^*f*/*f *^yolk sac tissues revealed delamination of the endodermal and mesodermal components of the yolk sac layers (Fig. 9C and 9D). Tissues immunolabelled by the VEC marker PECAM-1 showed separation of the endodermal and mesodermal tissues (compare Fig. 9C and 9D, asterisks). Also, some areas of laminar separation were observed to contain blood cells, suggesting these areas derived from either blood vessels or blood islands. Quantitation of the linear length of laminar separation in cross-sections shows that yolk sac tissues from mutant animals have a greater degree of laminar separation than wild-type littermates (see Fig. 9E; 16.3 ± 10.8% vs. 62.0 ± 10.4% wild-type vs. mutant; p = 0.003).

### Mutant yolk sac tissues lack cell-cell junctions and intra-laminar collagen deposition

**Figure 10:**
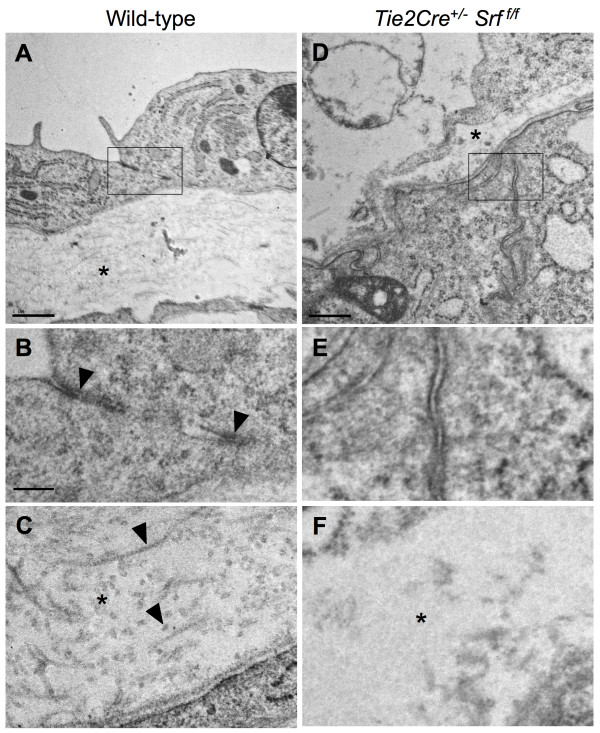
**Lack of junctional complexes and collagen deposition revealed by electron microscopy analysis of yolk sac tissues**. Yolk sac tissues from wild-type (A, B, C) and *Tie2Cre*^+/-^*Srf*^*f*/*f *^(D, E, F) embryos were subjected to transmission electron microscopy to assess ultrastructural detail. Desmosomal-type junctional complexes are observed in wild-type tissue (B arrowheads) but not in mutant tissue (E). Intralaminar collagen deposition occurs normally between endodermal and mesodermal cell layers in wild-type (A, C) but not mutant (D, F) tissues. Arrowheads in C denote longitudinal and cross-sectional views of individual collagen fibrils. Boxes in A and D correspond to images in B and E, respectively; asterisks in A and D correspond to images in C and F, respectively; scale bars: A, D = 1 μm; B, C, E, F = 0.2 μm.

Samples of yolk sac tissues from wild-type and *Tie2Cre*^+/-^*Srf*^*f*/*f *^embryos were processed for analysis by transmission electron microscopy (see Figure 10). Wild-type tissues contained desmosomal-type junction complexes that were absent in *Tie2Cre*^+/-^*Srf*^*f*/*f *^tissues (Fig. 10B, arrowheads; compare to Fig. 10E). Furthermore, collagen deposition in the extracellular matrix between the endodermal and mesodermal yolk sac layers was greatly diminished (Fig. 10C, arrowheads; compare to Fig. 10F). Sub-cellular elements of both endodermal and mesodermal cells in mutant tissue appeared largely normal, and did not display signs of necrosis nor apoptotic bodies. This observation suggests that the loss of cell-cell adhesion complexes is not due to gross disintegration of the yolk sac tissue as a whole, but rather is a consequence of SRF deprivation.

### E-Cadherin is decreased in *Tie2Cre*^+/-^*Srf*^*f/f *^embryonic yolk sac tissues

**Figure 11:**
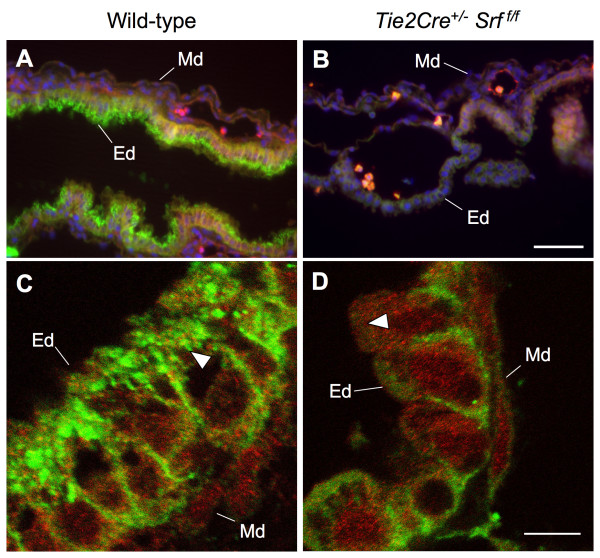
**E-Cadherin localization is disrupted in yolk sac tissues of *Tie2Cre*^+/-^*Srf*^*f*/*f *^embryos**. Photomicrographs of wild-type (A, C; 400×) and mutant (B, D; 2,000×) yolk sac tissues stained for PECAM (red) and E-cadherin (green). Immunofluorescent analysis revealed a decrease in detectable E-cadherin protein in mutant samples. Robust E-cadherin staining is observed in the endodermal (Ed) but not mesodermal (Md) layer of wild-type yolk sac (A). In contrast, very little E-cadherin staining can be detected in yolk sac from *Tie2Cre*^+/-^*Srf*^*f*/*f *^embryos (B). Single-plane confocal microscopy (C, D; 4,000×) confirms the lack of E-cadherin immunostaining at the apical brush-border surface of mutant yolk sac endodermal cells (compare A and B, arrowheads). Blue stain in A and B is DAPI nuclear stain. Scale bars: A, B = 50 μm; C, D = 7.5 μm.

We sought to confirm our electron microscopy observations by immunofluorescent labelling of junctional complexes by E-cadherin (see Figure 11). Wild-type and *Tie2Cre*^+/-^*Srf*^*f*/*f *^yolk sac tissues were double-labelled with the endothelial cell marker PECAM-1 and E-cadherin and subjected to fluorescent microscopy for analysis. Wild-type endodermal cells displayed intense E-cadherin localization along the apical brush-border surface, a staining pattern largely absent from mutant tissues (compare Fig. 11A and 11B). Closer examination by confocal laser microscopy confirmed the lack of detectable E-cadherin (compare Fig. 11C and 11D, arrowheads).

## Discussion

Numerous studies have shown that SRF is a critical transcriptional regulator of genes important for VSMC differentiation and development, and therefore a key regulator of vascular development. Consistent with this, SRF is required for differentiation of PE-derived precursors to VSMC during coronary vascular development [22]. We have previously shown, however, that SRF is expressed in the PE prior to detectable expression of VSMC markers [21], raising the possibility that SRF may play a role in differentiation of PE-derived coronary VEC as well. To begin to address this idea, we have sought to investigate the role of SRF in endothelial cells *in vivo*, and whether endothelial SRF is required for normal vascular development. Towards that end, we crossed mice expressing Cre recombinase under the VEC-specific *Tie2*-derived promoter with mice carrying floxed *Srf *alleles. Our data demonstrate that SRF plays a crucial role in appropriate VEC function beginning early in embryonic development.

Mutant embryos lacking SRF expression in VEC die by E14.5. Examination of heart structure reveals that hearts of *Tie2Cre*^+/-^*Srf*^*f*/*f *^embryos undergo looping and chamber formation; however, hearts of mutant embryos are measurably smaller with increasing gestational age compared to wild-type littermates. We did not detect a difference in the percentage of cardiomyocytes in heart tissue between wild-type and mutant embryos despite noting a small decrease in cardiomyocyte proliferation. Analysis of apoptotic activity by cleaved caspase 3 immunoreactivity also showed no discernible difference between wild-type and mutant embryos. Based on the well-documented specificity of the *Tie2Cre *driver, it is unlikely that the small decrease in cardiomyocyte proliferation observed is due to direct Cre-mediated excision of the *Srf *allele in cardiomyocytes [27]. One possible explanation for the reduction in cardiomyocyte proliferation in mutant embryos is that there is a defect in signalling to cardiomyocytes. In support of this idea, a variety of studies have demonstrated the importance of reciprocal signalling pathways between endocardial endothelial cells and the developing myocardium (for review see [29]). This raises the possibility that impaired signalling from SRF-null endocardial endothelial cells is contributing to reduced cardiomyocyte proliferation and the overall decrease in heart size observed in *Tie2Cre*^+/-^*Srf*^*f*/*f *^embryos. The apparent reduction in endocardial cell proliferation we observed in mutant embryos further suggests that loss of SRF in endothelial cells results in impaired endocardial endothelial cell function; however, which endothelial signalling pathways are impaired in SRF-null cells remains to be determined.

Endocardial cushion tissues are present; however, septal tissues appear poorly formed in mutant embryos, and in some cases exhibit a failure to form ventricular septa. Nevertheless, *in utero *lethality lacking gross defects in heart structure supports the idea of vascular failure as a cause of death. Lethal vessel malfunction may be occurring within the atrial tree, embryonic or extra-embryonic vasculature, or yolk sac vasculature. Consistent with this, mutant embryos exhibit hemorrhaging within the head, abdomen, and limbs as early as E12.5, a time when blood vessel remodelling is occurring. Yolk sac vasculature is also disrupted, suggesting a common mechanism of vessel malfunction in both embryonic and extra-embryonic tissues.

Initial vessel formation in the heart begins at approximately E11.5 and is completed with establishment of mature coronary arteries by E16.5 [28]. Early coronary vasculogenesis appears to occur normally in embryos lacking SRF in VEC as shown by the presence of PECAM-1-positive vessels in hearts from both wild-type and mutant E12.5 embryos. Nevertheless, mutant embryos die by E14.5, leaving the issue of SRF's role in coronary vasculature remodelling and maturation unclear. Determining the precise requirement for SRF in VEC specification is also complex since the *Tie2Cre *construct begins expression presumably after hemangioblast specification. Examination of blood smears from E12.5 embryos reveals no grossly observable differences between wild-type and mutant samples (data not shown). This may suggest that early VEC progenitors in the yolk sac are not influenced by excision of the *Srf *gene; however since the SRF protein has a relatively long half-life [30], we cannot rule out the possibility that residual SRF protein levels remain sufficient to sustain specification and differentiation of early VEC. These questions are better suited to investigation using *in vitro *model systems of VEC specification and differentiation, and are the object of ongoing studies.

While it is evident that SRF protein levels are reduced in VEC of mutant embryos, detectable SRF protein in both wild-type and *Tie2Cre*^+/-^*Srf*^*f*/*f *^embryos suggests a significant degree of mosaicism. Also, the stringency of our method for quantitation of SRF protein in VEC may result in an underestimation of the number of cells displaying detectable SRF protein. We have observed varying degrees of *Srf*-floxed allele excision efficiency based on the Cre recombinase driver, as well as possible variability from cell to cell. This results in a mosaic expression pattern between individual cells; however, the phenotypic effects of the excision still manifest in the tissue as a whole. Nevertheless, it is likely that more complete ablation may result in a more severe phenotype.

Yolk sac vascular networks undergo extensive remodelling during the course of embryogenesis. In our study, yolk sac vascular networks appear to form normally, echoing what was observed in initial coronary vessel development. However, yolk sac vascular networks became severely disrupted by E12.5, indicating a failure of the blood vessels to remodel and mature. Disintegration of yolk sac vasculature was accompanied by separation of endodermal and mesodermal cell layers. Further analysis revealed a lack of desmosomal junctions and intralaminar collagen as well as dramatic loss of E-cadherin in cells of the endodermal layer. Anchoring-type desmosomal junctions are plentiful in tissues subject to persistent mechanical stress, such as yolk sac, skin, and heart. Proper junction formation relies on appropriate expression of cytoskeletal and cell surface anchoring proteins such as actins, cadherins, and integrins. Recent work in our laboratory has shown that SRF-null cardiomyocytes have decreased levels of a number of genes associated with cell adhesion and cytoskeletal function including integrins β1 and α9, tight junction protein ZO-1, and protocadherins 7 and 18 [10], raising the possibility that the same genes may be affected in SRF-null endothelial cells, resulting in deficient cell-cell contacts.

Previous research has shown cadherins are also required for pericyte-endothelial interactions during angiogenic sprouting [31]. Furthermore, VE-cadherin, a major component of adherens junctions between endothelial cells, has been shown to promote angiogenesis [32]. Consistent with these findings, putative SRF-binding sites have been identified in the VE-cadherin-2/protocadherin12 [33], integrin-β1, integrin-β1 binding protein 2, protocadherin7 and protocadherin18 genes [10]. These observations suggest the involvement of SRF in regulation of cell-cell contacts; however as of yet, no experimental studies have addressed whether SRF is a significant player in these interactions, or whether SRF is a regulator of these genes in VEC.

Our results also raise the possibility that SRF may play a role in periendothelial cell/pericyte recruitment and vessel stabilization via involvement in angiopoietin (Ang) signalling. PECAM staining of yolk sac from Ang1-null mice shows a loss of vascular integrity similar to that seen in our study [34]. Ang1, a member of the angiopoietin family of ligands [25], activates the Tie2 receptor by binding to it and inducing its tyrosine phosphorylation. Ang1 is generated by non-endothelial cells, such as VSMC and other vascular pericytes recruited to vessels. Early in development between E9 to E11, Ang1 is found most prominently in the heart myocardium surrounding the endocardium. Later, it becomes more widely distributed, most often detected in the mesenchyme surrounding developing vessels and in close association with endothelial cells. Ang1-dependent vessel stabilization occurs by supporting reciprocal interactions between the vascular endothelium, pericytes, and surrounding extracellular matrix and mesenchyme [35]. Tie2 mediates Ang1 signalling via several known signalling pathways, including the Akt, RhoA/Rac1, MAPK, and ERK1/2 pathways [25]. Since SRF is a known mediator of all these pathways [36-39], this suggests that a role for SRF in Tie-2 mediated angiogenic remodelling may also lie as a downstream effector in the Ang1/2 signalling cascade in endothelial cells.

The results presented here unequivocally establish SRF as a critical regulator of endothelial cell function *in vivo *and indicate that appropriate SRF protein expression is required for remodelling of vascular networks. However, these results do not directly address the role of SRF in endothelial specification and differentiation since the *Tie2Cre *construct is expressed in differentiated endothelial cells. Our results suggest that disruption of anchoring-type proteins may play a significant role in the observed vascular failure, although the precise underlying molecular mechanisms remain to be addressed. Development of reagents that allow functional analyses of SRF loss of in endothelial precursors as well as vessel maturation will be required to more fully address these questions.

## Conclusion

Our study provides the first *in vivo *experimental evidence of a role for SRF in VEC function during embryonic development. Mouse embryos lacking SRF expression in endothelial cells die mid-gestation due to apparent vascular insufficiency. Initial analysis suggests a lethal malfunction in angiogenic remodelling and vessel maintenance. The extent to which this malfunction is due to failed reciprocal signalling between VEC and surrounding mesenchyme or perhaps incomplete vascular pericyte recruitment remains to be determined.

## Methods

### Mice and Genotyping

The *Tie2Cre *and *Srf*^*f*/*f *^transgenic mouse lines have been previously described [27,40]. Previous work has demonstrated that expression of the *Tie2Cre *gene begins early at E7.5; expression has been noted in all endothelial and endocardial tissues as well as some hematopoietic tissues [27]. Embryos were generated by timed mating, designating embryonic day 0.5 (E0.5) as noon on the day a vaginal plug was observed. Genotyping was performed under standard protocols using genomic DNA isolated from embryonic yolk sac, amnionic membrane, or tail tissue. Primers used were: *Tie2Cre *fwd 5'-GTTCGCAAGAACCTGATGGACA-3' and rev 5'-CTAGAGCCTGTTTTGCACGTTC-3'; *Srf*^*f*/*f *^fwd 5'-TGCTTACTGGAAAGCTCATGG-3' and rev 5'-TGCTGGTTTGGmCATCAACT-3'; *HPRT *fwd 5'-AGCGCAAGTTGAATCTGC-3' and rev 5'-AGCGACAATCTACCAGAG-3'. All procedures were in compliance with the Institutional Animal Care and Use Committee of the Medical College of Wisconsin.

### Quantitation of embryonic total body and isolated heart weights

Embryos were harvested from timed pregnant females and placed individually on a clean glass slide for weight determination; excess buffer fluid was absorbed by wicking with tissues prior to weighing. After whole body weight was recorded, the heart was dissected out and weighed separately. Amnionic membrane was reserved for genotypic analysis.

### Histology, Immunohistochemistry, and Microscopy

Embryos were harvested from timed pregnant females, photographed, and then fixed in either 4% paraformaldehyde/PBS or Tris-buffered zinc fixative (0.1 M Tris pH7.4 with 3.2 mM calcium acetate, 22.8 mM zinc acetate, and 36.7 mM zinc chloride). Yolk sac tissues selected for whole mount immunostaining were processed as described [41]. After staining, tissues were photographed and further processed for paraffin-embedding. All other samples were processed for paraffin-embedding, and subsequent sections (7 μm) were used for either hematoxylin & eosin staining, alcian blue staining, immunohistochemistry or confocal microscopy as described [42,43]. Reagents utilized were: anti-E-cadherin (BD Bioscience, clone 36); anti-sarcomeric myosin (University of Iowa Developmental Studies Hybridoma Bank, clone MF-20); anti-PECAM-1 (BD Biosciences, clone MEC13.3); anti-phospho-Histone H3 (Millipore, Ser-10); anti-SRF (ProteinTech custom). Digital image capture was performed using a Nikon SM2100 microscope with a Nikon CoolPix995 camera (whole mount), Nikon Eclipse TE300 microscope with a SpotII digital camera (tissue sections), or a Leica TCS SP2 laser scanning confocal microscope imaging system (confocal imaging). For electron microscopy, embryos were harvested from timed pregnant females and fixed in glutaraldehyde/sodium arsenate buffer. Subsequent processing and image collection were performed by the Electron Microscopy Core Facility at the Medical College of Wisconsin.

## Authors' contributions

MLH and RPM contributed equally to this project.

## Supplementary Material

Additional file 1 **Mitotic marker phospho Histone H3 appears decreased in *Tie2Cre*^+/-^*Srf*^*f/f *^endocardial endothelial cells compared to wild-type littermates**. Single-plane confocal images (630×) of sections of E11.5 mouse embryonic ventricular tissue (A, B) immunostained for endocardial endothelial cells (PECAM-1; red), cardiomyocytes (MF-20; green), and proliferating cells (phospho Histone H3, PhH3; blue). (C) Quantitation of PhH3-positive nuclei in either endocardial endothelial cells or cardiomyocytes (total PhH3-positive cells immunolabelled by PECAM-1 or MF-20 over four visual fields). *Tie2Cre*^+/-^*Srf*^*f/f *^mutant embryos exhibited reduced PhH3 immunoreactivity in endocardial endothelial cells compared to wild-type embryos, indicating a decrease in proliferation (arrowheads; C, left graph). MF-20-positive cardiomyocytes in mutant tissue exhibit a mild decrease in PhH3 immunostaining (arrows; C, right graph), which is consistent with the apparent reduction in overall myocardial size. Decreased myocardial mass is likely a secondary effect of inadequate signalling from SRF-null endocardial endothelial cells rather than Cre-mediated direct excision of SRF in cardiomyocytes. Also, consistent with previous reports (Nelson et al., *Circ. Res. *2004), presumed epicardium is negative for PECAM-1 staining in both wild-type and mutant embryos (asterisks). Scale bar: A, B = 30 μm. Click here for file

Additional file 2 **Fewer endocardial endothelial cells express SRF protein in *Tie2Cre*^+/-^*Srf*^*f*/*f *^embryos compared to wild-type littermates, however the number of SRF-positive cardiomyocytes remains similar**. Single-plane confocal images (630×) of E11.5 mouse embryonic ventricular tissue (A, B) immunostained for endocardial endothelial cells (PECAM-1; red), cardiomyocytes (MF-20; green), and SRF (blue). (C) Number of SRF-positive nuclei in either endocardial endothelial cells or cardiomyocytes (total SRF-positive nuclei in either endocardial endothelial cells or cardiomyocytes over four visual fields). *Tie2Cre*^+/-^*Srf*^*f*/*f *^mutant embryos exhibited a reduced number of SRF-labelled nuclei in endocardial endothelial cells, consistent with a decrease in proliferation (arrowheads; C, right graph). MF-20-positive cardiomyocytes in mutant tissue exhibit virtually no change in SRF-labelled nuclei (arrows; C, left graph). These results are consistent with a signalling defect in SRF-null endocardial endothelial cells, and raise the possibility that the decrease in myocardial mass observed in mutant embryos may be related to inadequate reciprocal signalling between endocardial endothelial cells and the underlying myocardium rather than from Cre-mediated direct excision of the *Srf *gene in cardiomyocytes. Scale bar: 30 μm. Click here for file
