# Supplementary figures and images for: Endothelial-specific ablation of Serum Response Factor causes hemorrhaging, yolk sac vascular failure, and embryonic lethality
Source: BMC Dev Biol. 2008 Jun 20;8:65. doi: 10.1186/1471-213X-8-65 (PMC2442838; doi:10.1186/1471-213X-8-65)

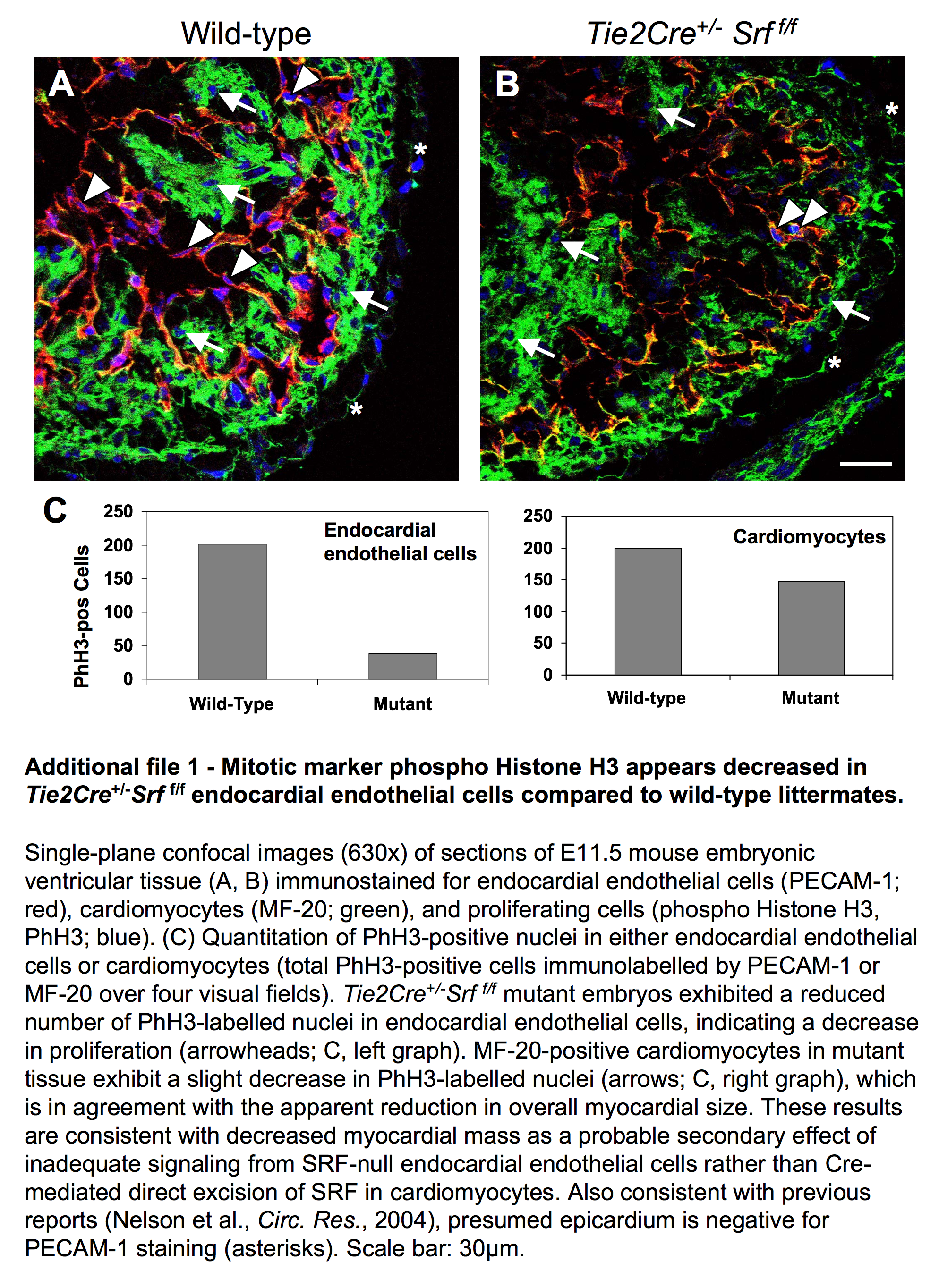

Supplement: Additional file 1 — Mitotic marker phospho Histone H3 appears decreased in Tie2Cre+/-Srff/f endocardial endothelial cells compared to wild-type littermates. Single-plane confocal images (630×) of sections of E11.5 mouse embryonic ventricular tissue (A, B) immunostained for endocardial endothelial cells (PECAM-1; red), cardiomyocytes (MF-20; green), and proliferating cells (phospho Histone H3, PhH3; blue). (C) Quantitation of PhH3-positive nuclei in either endocardial endothelial cells or cardiomyocytes (total PhH3-positive cells immunolabelled by PECAM-1 or MF-20 over four visual fields). Tie2Cre+/-Srff/f mutant embryos exhibited reduced PhH3 immunoreactivity in endocardial endothelial cells compared to wild-type embryos, indicating a decrease in proliferation (arrowheads; C, left graph). MF-20-positive cardiomyocytes in mutant tissue exhibit a mild decrease in PhH3 immunostaining (arrows; C, right graph), which is consistent with the apparent reduction in overall myocardial size. Decreased myocardial mass is likely a secondary effect of inadequate signalling from SRF-null endocardial endothelial cells rather than Cre-mediated direct excision of SRF in cardiomyocytes. Also, consistent with previous reports (Nelson et al., Circ. Res. 2004), presumed epicardium is negative for PECAM-1 staining in both wild-type and mutant embryos (asterisks). Scale bar: A, B = 30 μm. [file 1471-213X-8-65-S1.png]

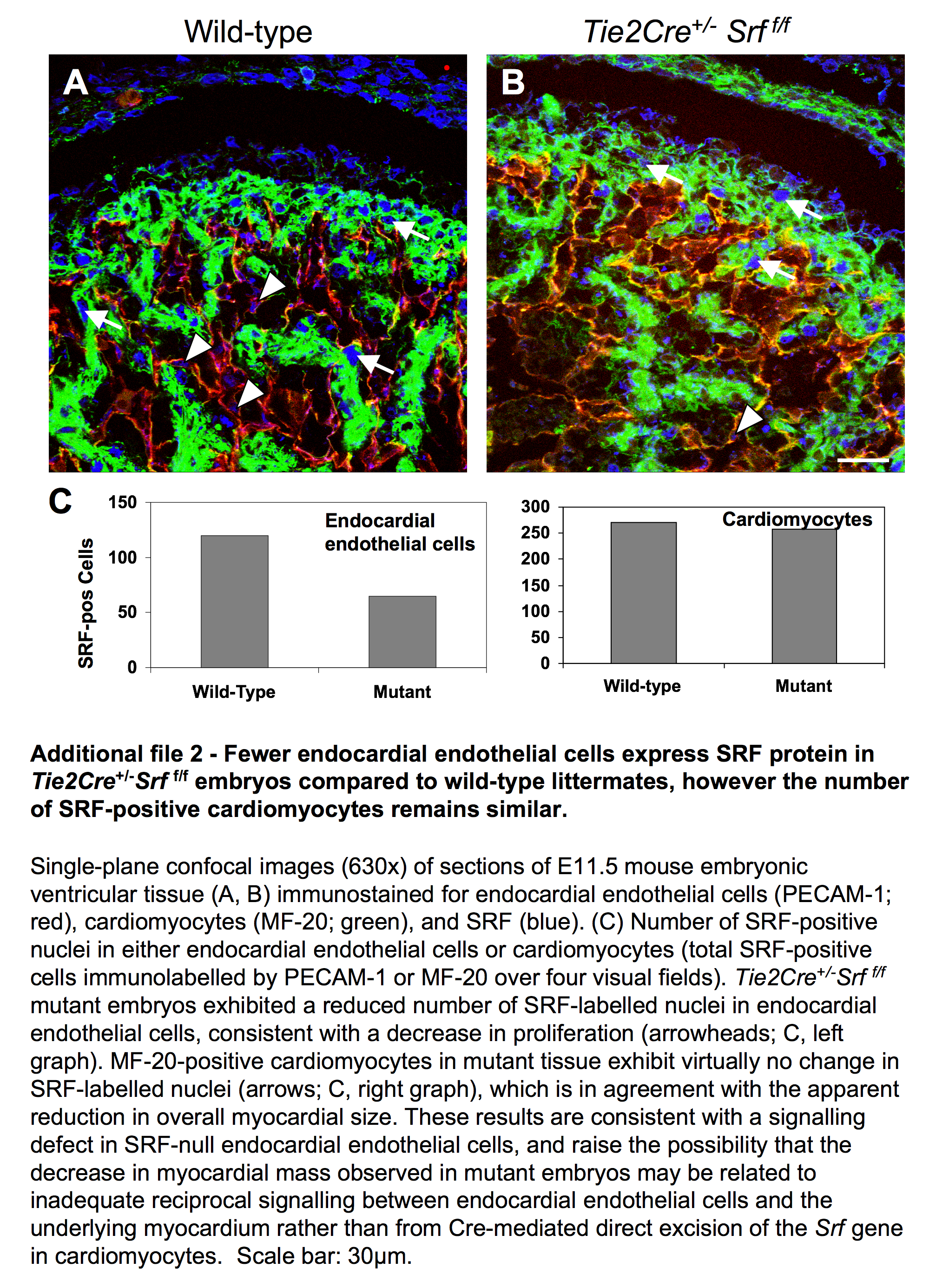

Supplement: Additional file 2 — Fewer endocardial endothelial cells express SRF protein in Tie2Cre+/-Srff/f embryos compared to wild-type littermates, however the number of SRF-positive cardiomyocytes remains similar. Single-plane confocal images (630×) of E11.5 mouse embryonic ventricular tissue (A, B) immunostained for endocardial endothelial cells (PECAM-1; red), cardiomyocytes (MF-20; green), and SRF (blue). (C) Number of SRF-positive nuclei in either endocardial endothelial cells or cardiomyocytes (total SRF-positive nuclei in either endocardial endothelial cells or cardiomyocytes over four visual fields). Tie2Cre+/-Srff/f mutant embryos exhibited a reduced number of SRF-labelled nuclei in endocardial endothelial cells, consistent with a decrease in proliferation (arrowheads; C, right graph). MF-20-positive cardiomyocytes in mutant tissue exhibit virtually no change in SRF-labelled nuclei (arrows; C, left graph). These results are consistent with a signalling defect in SRF-null endocardial endothelial cells, and raise the possibility that the decrease in myocardial mass observed in mutant embryos may be related to inadequate reciprocal signalling between endocardial endothelial cells and the underlying myocardium rather than from Cre-mediated direct excision of the Srf gene in cardiomyocytes. Scale bar: 30 μm. [file 1471-213X-8-65-S2.png]
